# Fully Phased Population‐Prevalent East African Cattle BoLA‐I Alleles Determined Using PacBio HiFi Long‐Read Sequencing Represent Five Novel Specificities With Distinctive Peptide Binding Potential

**DOI:** 10.1111/tan.70183

**Published:** 2025-04-17

**Authors:** Isaiah Obara, Andreotti Sandro, Khawla Elati, Timothy Conneley, Morten Nielsen, Naftaly Githaka, Anne Nanteza, Richard Bishop, Ard Nijhof

**Affiliations:** ^1^ Freie Universität Berlin Institute for Parasitology and Tropical Veterinary Medicine, Department of Veterinary Medicine Berlin Germany; ^2^ Freie Universität Berlin Veterinary Centre for Resistance Research, Department of Veterinary Medicine Berlin Germany; ^3^ Freie Universität Berlin Institute of Computer Science, Department of Mathematics and Computer Science Berlin Germany; ^4^ The Roslin Institute and Royal (Dick) School of Veterinary Studies University of Edinburgh Edinburgh UK; ^5^ Centre for Tropical Livestock Genetics and Health Roslin Institute, University of Edinburgh Edinburgh UK; ^6^ Department of Health Technology, Technical University of Denmark Kongens Lyngby Denmark; ^7^ The International Livestock Research Institute Nairobi Kenya; ^8^ Department of Biotechnical and Diagnostic Sciences, College of Veterinary Medicine, Animal Resources and Biosecurity (COVAB) Makerere University Kampala Uganda; ^9^ Department of Veterinary Microbiology and Pathology Washington State University Pullman Washington USA

**Keywords:** cattle, high‐throughput sequencing, MHC

## Abstract

Due to factors such as lower biosecurity, greater wildlife/farm animal interfaces, and environmental challenges, cattle in sub‐Saharan Africa are exposed to more diverse and intensive bacterial, viral and protozoan pathogen challenges than cattle in Europe and other high‐income regions of the world. Classical class I genes of the major histocompatibility complex (MHC) contribute to protection from diseases caused by these pathogens by refining a huge pool of potential pathogen‐derived peptide ligands into a smaller ensemble for presentation to CD8+ T cells. Knowledge of population‐prevalent MHC alleles is therefore critical for evidence‐based approaches to vaccine design and improved understanding of pathogen resistance. Whereas variation in MHC molecules is understood in most detail for European *Bos taurus* , the alleles expressed by Africa's cattle remain poorly defined. We have leveraged recent improvements in the accuracy of PacBio high‐fidelity (HiFi) circular consensus sequencing (CCS) and adapted stringent sequence filtering algorithms to identify hundreds of as yet uncharacterised fully phased BoLA‐I alleles from multiple populations of African taurine (Ankole) and indicine (Zebu) cattle in East Africa. The analysis highlights a convergence of population‐prevalent class I MHC allelic repertoires in taurine and indicine cattle, likely due to the similar pathogen‐driven selective pressures. Our analysis of the anchor residue accommodating pockets of these prevalent alleles revealed extremely high levels of polymorphism, which contrast with Holstein alleles that exhibit a more limited repertoire of MHC specificity‐determining pocket residues, potentially constraining the breadth of peptide presentation. However, in the context of considerable sequence and physicochemical variation in the pocket‐forming residues, it was possible to discern overlaps in the predicted peptide binding spectrum. Interrogation of potential differences in peptide binding specificities with European *B. taurus* alleles revealed that the fully phased African cattle class I MHC alleles represent five novel specificities. We envisage that this novel finding will find broad application in assessing potentially achievable vaccination coverages of future pathogen‐encoded vaccine candidates against important intracellular pathogens. One aim of future research should be to leverage recent improvements in the sensitivity of mass spectrometry combined with immunoprecipitation of peptides bound to African cattle MHC to search directly for T‐cell epitopes in the context of the inferred ‘supertype’ diversity.

## 1 Introduction

The major histocompatibility complex (MHC) is a region within higher vertebrate genomes that comprises multiple, closely linked, polymorphic loci with a variety of immunological functions [1, 2]. Genes within this complex encode cell surface proteins which can be grouped into three distinct clusters: class I, II and III according to their function. Class I MHC gene products are present on the surface of every nucleated cell and are crucial to defence against intracellular pathogens by presenting pathogen‐derived peptide fragments at the cell surface where they can be surveyed by antigen‐specific CD8+ T cells, potentially triggering their clonal expansion and differentiation into effector and memory cells. Similarly, peptide‐MHC I complexes on cell surfaces are also recognised by various receptors expressed by NK cells that play a key role in regulating their diverse functions.

There remain significant gaps in the knowledge of allelic polymorphism and sequence divergence across the classical genes of the MHC complex in African cattle, and this constrains infectious disease research and vaccine development. African cattle have their origins in several geographically separated genotypes of the now extinct wild ox or Auroch ( *Bos primigenius* ) that inhabited the forests and plains of Europe, Asia and possibly North Africa [3, 4]. They are a genetically diverse population that has generally resulted from the successive introduction of Asian *Bos indicus* and European *Bos taurus* cattle over the last 2000 years. Given these complex diverse origins from multiple ancestral populations, immunogenetic diversity at the MHC loci may reflect variations within the progenitor wild ox populations or in a more distant ancestor. In addition, a molecular arms race with pathogens is known to impose significant selection pressure on classical MHC molecules and drive MHC allele frequency changes in populations. Africa's cattle have had to survive a very high diversity and intensity of pathogen challenge because exposure to bacterial, viral and protozoan pathogens is greater in cattle in sub‐Saharan Africa than Eurasia [5].

Although an array of pathogens will contribute to the total selective pressure experienced by African cattle, it has become apparent that single complex pathogens like the apicomplexan parasite *Theileria parva* may have a particularly strong effect on host fitness [6]. *T. parva* immortalises bovine lymphocytes resulting in an acute lymphocytic tumour‐like disease termed East Coast fever (ECF) that kills 1 million cattle annually [7]. A number of studies have demonstrated that class I MHC restricted cytotoxic T lymphocytes (CTLs) form the basis of protective immune responses following live vaccination for control of this parasite. As the adaptive interface of pathogen recognition, it is likely the class I MHC peptide binding motifs continuously adapt to optimise peptide presentation as the pathogen antigens evolve. Such pathogen‐induced immune adaptation is likely responsible for the susceptibility spectrum seen across domestic and wild animal species. At one extreme of the spectrum are the non‐indigenous Holstein cattle ( *B. taurus* ) introduced into Africa over the last 100 years, which often succumb to *T. parva* infection, even in well‐resourced ranches, despite widespread use of acaricidal dipping. Indigenous African taurine and indicine cattle that live under endemic disease challenge exhibit varying levels of tolerance. Given that MHC genes evolve rapidly, the potential for ongoing selection of host MHC by *T. parva* is therefore greatest in African cattle present in the *T. parva* endemic area, which extends over a wide region of 17 countries from Sudan in the North to South Africa in the South [8].

As opposed to the European Holstein/Friesian ( *B. taurus* ) cattle, where most class MHC alleles have been identified (www.ebi.ac.uk/ipd/mhc/bola) [9], African cattle MHC remains poorly characterised. This has constrained rational identification of the repertoire of bound and presented peptides that will likely be required for the development of multivalent vaccines that can potentially overcome the issue of the histocompatibility barrier in the field. For example, to date, only a limited number of Holstein cattle, with a very limited range of class I MHC alleles, have been examined for the antigenic specificity of *T. parva* ‐specific CD8+ T cells. However, the current suite of Holstein CD8+ T‐cell targets are not recognised by immune CD8+ T cells from African taurine and Indicine cattle in endemic areas, suggesting class I MHC functional divergence between these cattle populations [10, 11]. In fact, it has become increasingly evident that individual hosts of different class I MHC genotypes generally respond to different sets of epitopes, often in different proteins.

We sought herein to understand what might be novel and distinctive about the class I MHC genes in African taurine and indicine cattle in *T. parva* endemic areas. The main hallmarks of the MHC—the dense polymorphism and existence of multiple loci—constrain efficient and reliable genotyping. For example, cattle have at least six classical class I MHC genes (contrasting with three in humans) that share high sequence similarities, making it impossible to design gene‐specific PCR primers for these genes [12, 13, 14]. In addition, certain cattle class I MHC alleles differ from one another by as few as 1 bp and therefore require a genotyping approach with exceptionally high resolution to segregate. Also, there is a variable class I MHC haplotype structure in cattle, with anywhere between 1 and 4 classical class I genes present and expressed, in a variety of combinations (https://www.ebi.ac.uk/ipd/mhc/group/BoLA/haplotype/), which makes the number of alleles per individual highly variable and difficult to predict.

While next‐generation sequencing (NGS) technologies have proved effective for precise typing of MHC alleles, the utility of the NGS approaches used so far to study allelic heterogeneity at the bovine MHC has been impeded majorly by their short‐read lengths. For example, Illumina read lengths are insufficient to allow phasing of exon 2 and 3‐derived sequences, so these are typically analysed separately or by assembly of partial sequences generated from two separate amplicons, which can potentially introduce assembly errors [15, 16, 17]. Furthermore, most sequences available in public databases represent only incomplete exon 2 fragments, as the forward primers are often located within this exon. As such, these sequences fail to meet the requirements of in silico analysis aimed at predicting peptides with a higher probability of being immunodominant on the basis of their predicted MHC binding affinity, which requires the complete second and third exon sequences.

We leveraged the improved accuracy of PacBio high‐fidelity (HiFi) circular consensus sequence (CCS) base calls (99.9%), made possible by recent technological improvements, to provide novel insights into African cattle immunogenetics. The PacBio sequel II HiFi long read based sequencing of pooled cDNA amplicons, coupled with stringent sequence filtering algorithms for disaggregating legitimate variants from artefacts, produced fully phased class I MHC alleles spanning multiple functional domains. This high‐resolution population‐scale MHC typing in African taurine and indicine cattle enabled identification of population‐prevalent alleles in African taurine and indicine cattle that represent at least five novel predicted peptide binding specificities when compared to European cattle alleles.

## 2 Materials and Methods

### 2.1 Taurine and Indicine Cattle RNA Samples

**FIGURE 1:**
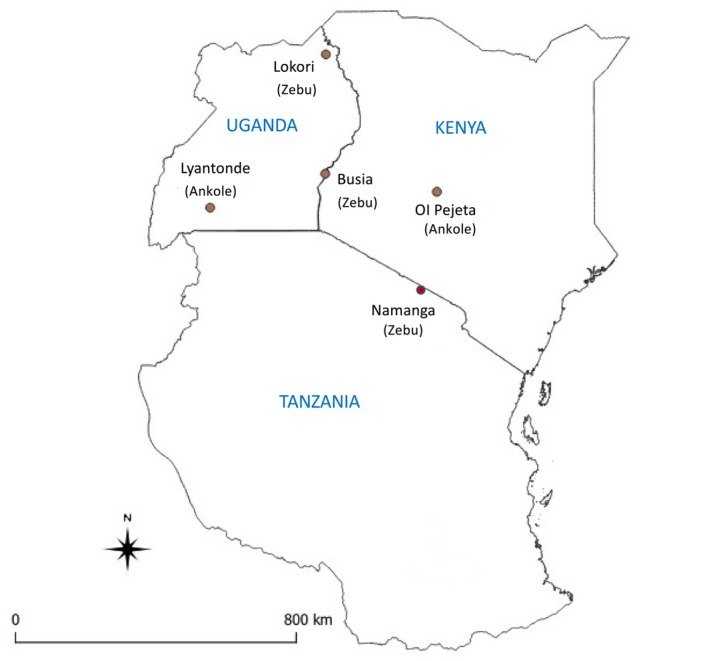
Map of Kenya, Uganda and Tanzania showing the location of sampling sites.

Tissue samples (ear notches) were obtained from different Zebu ( *B. indicus* ) and Ankole ( *B. taurus* ) populations from five locations across Kenya, Uganda and Tanzania, as shown in Figure 1. A total of 300 samples were collected from the five locations shown in the map in Figure 1. The cattle were sampled according to standard operating procedures approved by the Kenya National Commission for Science, Technology and Innovation (NACOSTI), Makerere University, College of Veterinary Medicine, Animal Resources and Bio‐security (COVAB) and College of Veterinary Medicine and Biomedical Sciences of Sokoine University of Agriculture, Tanzania. Licence numbers: NACOSTI/P/23/24659 and COVAB/COD/96/05. The tissue samples were stored in RNAlater prior to RNA extraction. Fine grinding of the tissues was achieved by agitation in a high‐speed bead mill using 4.7 mm steel beads optimised for disruption of hard samples (Innuscreen, Germany). Total cellular RNA was isolated from the homogenised tissues using the NucleoSpin RNA isolation kit (Macherey‐Nagel, Germany), according to the manufacturer's instructions. The purified RNA was used as a template for Oligo dt‐primed cDNA synthesis using the ProtoScript II First Strand cDNA Synthesis System (New England Biolabs).

### 2.2 Amplification of Full‐Length Bovine Class I MHC Transcripts

Degenerate oligonucleotide primers, 5'‐ATGRGGCCGCGARCCCTC‐3' and 5'‐TGMGAGACACATCAGAGCCC‐3', based on the most conserved sequences present in all publicly available cattle class I MHC sequence databases (and additionally conserved between cattle and the European Bison) were used. The Bison is classified within the same tribe as cattle, but in a different genus [18]. These primers amplify exons 1–7 of the class I MHC heavy chain. To link multiplexed sequence reads to the original samples, a unique 16‐bp index sequence was added to the 5' end of each primer. The asymmetric barcoding strategy used involved amplification of samples using a unique combination of one of eight barcoded forward primers and one of 24 barcoded reverse primers, allowing recovery of amplicons from hundreds of pooled individuals in a single PacBio sequel II HiFi sequencing run. Besides the index and the degenerate gene‐specific sequences, each primer also contained a 5' spacer sequence (GCATC) and a 5' modification to prevent amplicons without barcodes forming SMRTbell templates during library construction. The primer sequences used for asymmetric barcoding are listed in Table 1.

**TABLE 1:** Oligonucleotide primers used in the asymmetric barcoding strategy for amplification and PacBio sequel II HiFi sequencing of African cattle class I MHC.

Barcoded forward primers			
5' Modfication	Spacer	Barcode	Degenerate forward primer
[Phos]	GCATC	CACTCGACTCTCGCGT	ATGRGGCCGCGARCCCTC
[Phos]	GCATC	TCTGTATCTCTATGTG	ATGRGGCCGCGARCCCTC
[Phos]	GCATC	ACAGTCGAGCGCTGCG	ATGRGGCCGCGARCCCTC
[Phos]	GCATC	ACACTAGATCGCGTGT	ATGRGGCCGCGARCCCTC
[Phos]	GCATC	CGCATGACACGTGTGT	ATGRGGCCGCGARCCCTC
[Phos]	GCATC	CACGACACGACGATGT	ATGRGGCCGCGARCCCTC
[Phos]	GCATC	CACTCACGTGTGATAT	ATGRGGCCGCGARCCCTC
[Phos]	GCATC	CATGTAGAGCAGAGAG	ATGRGGCCGCGARCCCTC

Barcoded reverse primers			
5' Modfication	Spacer	Barcode	Degenerate reverse primer
[Phos] [Phos] [Phos] [Phos] [Phos] [Phos] [Phos] [Phos] [Phos] [Phos] [Phos] [Phos] [Phos] [Phos] [Phos] [Phos] [Phos] [Phos] [Phos] [Phos] [Phos] [Phos] [Phos] [Phos]	GCATC GCATC GCATC GCATC GCATC GCATC GCATC GCATC GCATC GCATC GCATC GCATC GCATC GCATC GCATC GCATC GCATC GCATC GCATC GCATC GCATC GCATC GCATC GCATC	AGAGACTGCGACGAGA CAGAGAGTGCGCGCGC CGCGCGTCGTCTCAGC AGAGAGTACGATATGT TCTGTAGTGCGTGCGC ATGTGCGTGTGTGTCT CTCTCAGACGCTCGTC TATCTCAGTGCGTGTG TGTGTCTATACTCATC TATAGACTATCTGAGA GTATGTGAGAGAGCGC CACGCGACGCTCTCTA GAGAGCGCGAGTGCAC GTGCTCTGTGTGTCAC TGCGTGTATGTCATAT ACGAGATACTCGCGCG CTGTGTAGAGAGCACA TGATGTGACACTGCGC ACTACTGAGACATAGA TATATCGCGTCGCTAT GCGTACTGCGACTGTG ATATATGCACGCTCTA CGCTGTATACACGCTC AGAGACTGTAGCGCAC	TGMGAGACACATCAGAGCCC TGMGAGACACATCAGAGCCC TGMGAGACACATCAGAGCCC TGMGAGACACATCAGAGCCC TGMGAGACACATCAGAGCCC TGMGAGACACATCAGAGCCC TGMGAGACACATCAGAGCCC TGMGAGACACATCAGAGCCC TGMGAGACACATCAGAGCCC TGMGAGACACATCAGAGCCC TGMGAGACACATCAGAGCCC TGMGAGACACATCAGAGCCC TGMGAGACACATCAGAGCCC TGMGAGACACATCAGAGCCC TGMGAGACACATCAGAGCCC TGMGAGACACATCAGAGCCC TGMGAGACACATCAGAGCCC TGMGAGACACATCAGAGCCC TGMGAGACACATCAGAGCCC TGMGAGACACATCAGAGCCC TGMGAGACACATCAGAGCCC TGMGAGACACATCAGAGCCC TGMGAGACACATCAGAGCCC TGMGAGACACATCAGAGCCC

For PCR amplfications, we used Platinum SuperFi II PCR Master Mix (Invitrogen). The cycling conditions for PCR were 98°C for 30 s, 30 cycles of 98°C for 10 s, 60°C for 10 s, 72°C for 30 s and a final extension of 72°C for 5 min. The products of each PCR reaction were separated on a 1% agarose gel, stained with gel red and visualised under a UV transilluminator. The PCR products were purified using the PCR Clean‐Up System (Promega), and quantified using a Nanodrop (ThermoScientific).

The class I MHC amplicons were pooled proportionately to their concentrations and submitted to the Biomarker Technologies (BMKGENE) sequencing facility in Münster, Germany, where the amplicons were initially quality controlled to ascertain the size, concentration and amount of DNA. Once pools pass these checks, they enter the SMRTbell library preparation pipeline using the PacBio HiFi protocols. Sequencing was performed using a PacBio Sequel II SMRT Cell in HiFi mode.

### 2.3 Sequence Data Processing and Allele Calling

Starting from bam files containing raw subreads, the program CCS was used for the generation of CCSs with a filter set to retain only CCS reads making five or greater full passes around the closed loop SMRTbell amplicon (~99.9% accuracy). The program lima (version 2.9.0) was used to curate the CCS reads and separate them by barcode. These initial steps in the workflow were implemented in the SMRT LINK 13.1 software package (https://www.pacb.com/support/software‐downloads/).

To ‘denoise’ the CCS reads, we initially used DADA2 (version 1.26.0) the successor of the Divisive Amplicon Denoising Algorithm, an approach that learns and parameterises a dataset‐specific error model rather than imposing arbitrary dissimilarity thresholds or relying on previous datasets that may not reflect the PCR and sequencing protocols specific for a given study. Details of the steps of the DADA2 pipeline can be found in [19]. Briefly, DADA2 takes as input amplicon sequence data in fastq files and subjects these to primer trimming, orienting the CCS reads in the forward direction, quality filtering, dereplication, learning the dataset‐specific error model, inferring putative alleles, and identifying and discarding chimeras. We used stringent DADA2 settings requiring that primers map without any mismatches, minimum quality base (minQ) set to 3 and the maximum expected errors for the complete trimmed read set to 1. We also used a length filter of 800 < = length < = 1200.

We chose to augment the DADA2 workflow by applying additional filters as described in [20]. First, all known full‐length cattle class I MHC alleles were downloaded from the IPD/MHC/BoLA database. This reference set of curated nucleotide and protein sequences was used to set up local BLAST databases. The DADA2 filter‐pass variants were translated to detect frameshifts and premature stop codons, and those shorter than 330 amino acids (AA) discarded. We undertook blastp (version 2.14.0) searches of the retained variants against the reference alleles in the local BLAST database, and those with less than 60% AA identity and < 90% query coverage were also discarded [21]. Then, based on all pairwise alignments using mmseqs2 (version 14.7e284) [22], we identified and discarded variants that were within two mismatches from another allele (of higher abundance) and appeared only in a single sample. Finally, we filtered alleles that matched (blastn, version 2.14.0, 98% identity, 99% query coverage) to one of the known bovine non‐classical class I MHC alleles. The allelic sequences identified were submitted to GenBank (ID: 2857708).

### 2.4 Analysis of Selection Pressures Acting on the African Cattle Class I MHC

We used the Single‐likelihood Ancestor Counting (SLAC) method incorporated in the Hyphy package (Kosakovsky Pond et al. 2020) within the Datamonkey interface (Delport et al. 2010) to detect signatures of selection by the rate of non‐synonymous to synonymous substitutions in BoLA‐I alleles. SLAC uses a combination of maximum‐likelihood and counting approaches to infer nonsynonymous and synonymous substitution rates on a per‐site basis and requires a coding alignment and corresponding phylogeny as input (Kosakovsky Pond et al. 2005). The branch lengths and nucleotide substitution parameters were optimised under the MG94xREV model and the Akaike information criterion (AIC) was used to evaluate the fit of the model to the MHC dataset. At each site where positive selection was inferred, significance was ascertained using an extended binomial distribution.

### 2.5 In Silico Analysis of African Cattle Class I MHC Peptide Binding Potential

CTLs are triggered through proteolytic processing of antigens derived from intracellular pathogens, resulting in a large pool of potential peptide ligands that are filtered into a smaller ensemble based on binding with high affinity to class I MHC genes. To understand the potential functional consequences of African cattle class I MHC variation on peptide selection for immune recognition, we clustered the African cattle class I MHC alleles into groups with differing peptide binding potential at their antigen‐binding sites. This analysis required use of the machine learning neural network predictor NetMHCPan‐4.1, a version trained on immunopeptidomic and binding affinity datasets [23]. The functional distance between alleles was derived from correlations between their NetMHCpan‐4.1 predicted peptide binding affinities and used to generate a functional distance tree, where groups of putatively functionally similar MHC variants cluster together.

## 3 Results

### 3.1 Circular Consensus Class I MHC Sequence Reads

**FIGURE 2:**
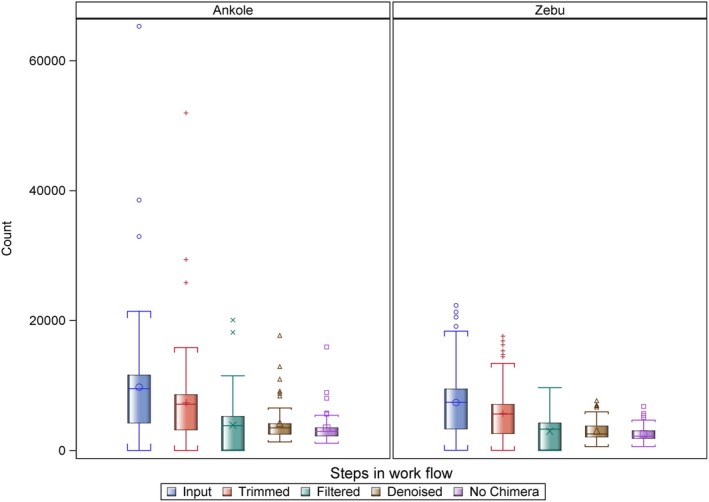
Box plot displaying the distribution of retained CCS reads, grouped by breed, following a stepwise filtering criterion. ‘Trimmed’ refers to CCS reads where both primers were detected without any mismatches and trimmed. ‘Filtered’ represent retained CCS reads upon filtering based on the DADA2 algorithm using minQ (min quality base) 3 and maxEE (expected errors for the complete trimmed read) = 1 and 800 < = length < = 1200. CCS reads that could be assigned to the inferred amplicon sequence variants are marked as ‘Denoised’, while ‘nochim’ denotes reads retained following application of the DADA2 chimera detection and removal algorithm. 76%, 40%, 29% and 24% of reads were retained after the trimming, filtering, denoising and chimera removal steps, respectively.

PacBio HiFi sequencing generated 240,275,911 sub‐reads with a total of 323,132,188,565 bp. The sub‐reads were used for the generation of 1,840,768 CCSs with a total of 2,851,646,167 bp. A total of 1,456,345 CCS reads were retained following the application of the 800 < = length < = 1200 read‐length filter. We implemented DADA2, a version specifically designed to infer amplicon sequence variants from long amplicon read datasets with high‐quality scores, as described in the Materials and Methods. As already mentioned, additional filtering was based on the methods described by Sommer et al. (2013). Figure 2 summarises the results of the stepwise sequence filtering process that excluded reads categorised as of insufficient quality and also disaggregated putative alleles from artefactual sequences and chimeras. The mean number of per animal filter‐pass reads was 2982.

### 3.2 Considerable Pairwise Sequence Dissimilarity Among the Fully Phased Class I MHC Transcripts

**FIGURE 3:**
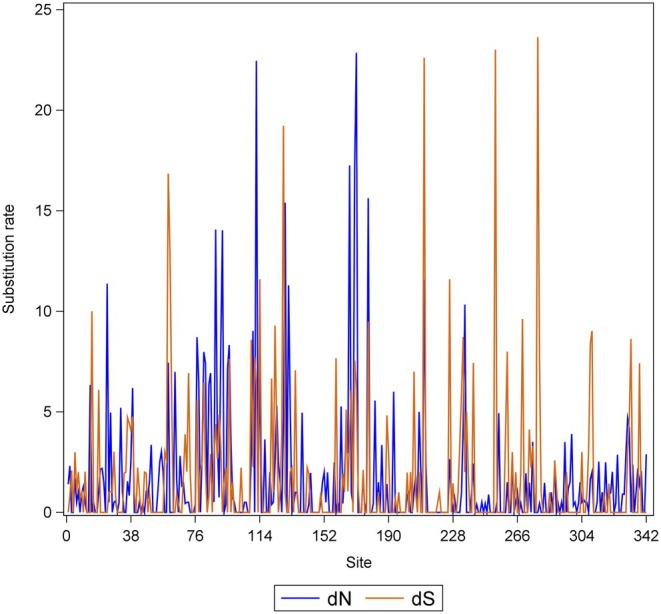
A site graph of the inferred rates of synonymous (orange) and non‐synonymous (blue) substitutions for African cattle BoLA‐I alleles. In this figure, the defined BoLA‐I domains include alpha (*α*) 1 (residues at sites 16–106), *α*2 (107–198), *α*3 (199–290), transmembrane (291–324) and an intra‐cytoplasmic (325–342).

Analysis of the trimmed and filtered class I MHC transcripts revealed a 1026‐nucleotide open reading frame predicted to encode a 342‐amino acid protein. The full‐length class I MHC transcript contains a 1077‐nucleotide open reading frame that encodes a 358‐amino acid protein. Although useful for accurate phylogenetic designation, the few codons missing from the amplicon are located in the intra‐cytoplasmic domain and therefore of little relevance from a functional peptide‐binding perspective. Among the 1026 nucleotide sites, 444 sites (43.27%) were found to be polymorphic. The deduced amino acid sequences of alleles at the African cattle class I MHC loci show considerable diversity relative to the nucleotide sequences indicating that a majority of the mutations are amino acid altering. Indeed, our analysis revealed evidence for the action of positive selection at 28 sites at *p* ≤ 0.1. As expected, these are located predominantly within the peptide binding region. At the same *p* value threshold, 36 sites, mostly outside the peptide binding region, were identified as being under negative/purifying selection. A site graph of the inferred rates of synonymous and non‐synonymous substitutions is shown in Figure 3.

### 3.3 Population‐Prevalent African Cattle Class I MHC Alleles

**FIGURE 4:**
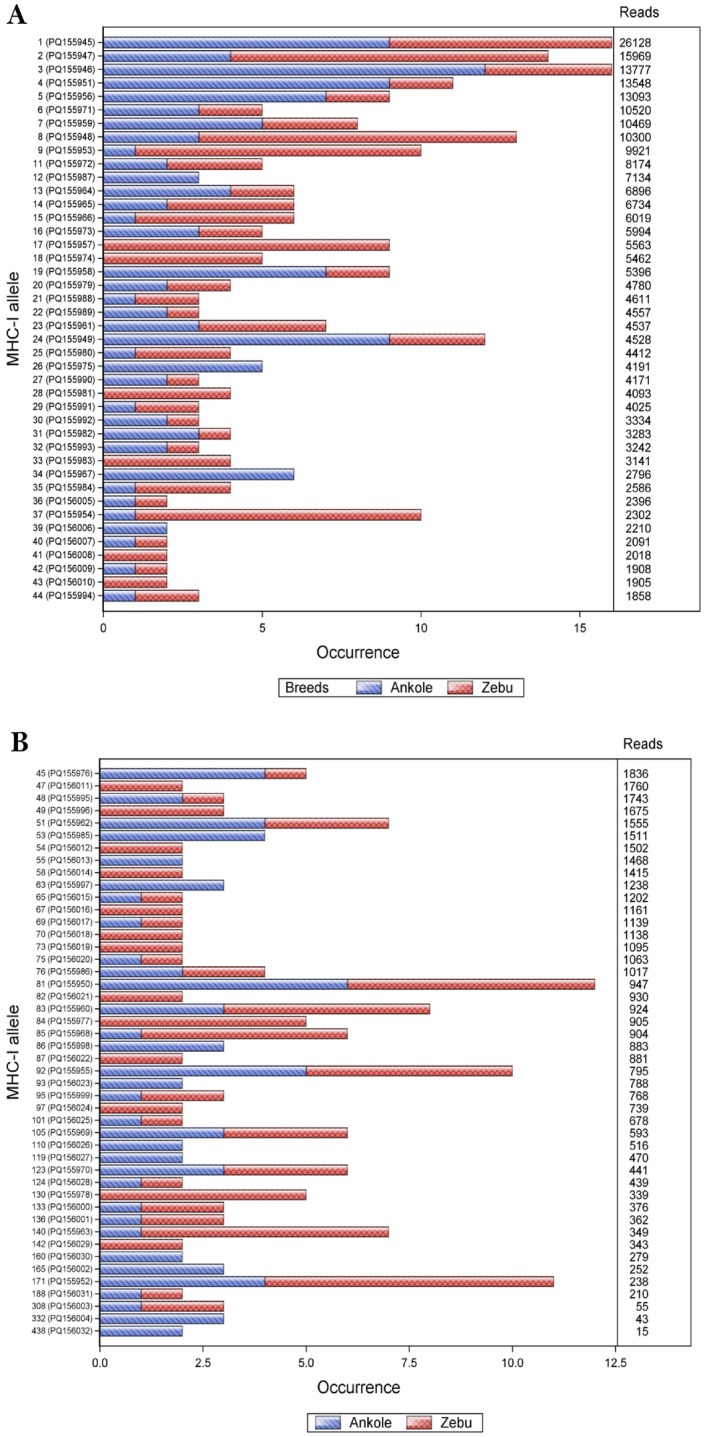
Population‐prevalent African cattle class I MHC alleles grouped by breed. The authors' provisional allelic designations and their accession numbers in parentheses for the 87 population‐prevalent alleles are shown on the *y*‐axis. The occurrence of each of these alleles, grouped by breed, is shown on the *x*‐axis. The number of filter‐pass reads supporting each allele is shown to the right.

Out of the 250 variants that were retained following the stepwise filtering criterion described above, 87 were transcribed in at least two animals, with some being present in up to 18 animals, thereby meeting the requirement for inclusion in IPD‐MHC, which is the official repository for annotated and curated non‐human MHC sequences. These are hereafter referred to as ‘population‐prevalent alleles,’ and as shown in Figure 4, their occurrence in Ankole and Zebu cattle highlights a convergence of BoLA‐I allelic repertoires in the two breeds. Also shown in Figure 4 is the number of filter‐pass reads supporting each allele.

An initial indication of the allele content of African cattle class I MHC haplotypes based on comparisons of the subset of alleles transcribed in individual animals is provided in Table 2.

**TABLE 2:** Alleles that contribute to putative African cattle class I MHC haplotypes.

Allele content of African cattle I MHC haplotypes				
Allele 1	Allele 2	Allele 3	Allele 4	Support (no. of animals)
1 1 2 3 11 18 11 1 24 1 1 2 3 3 3 8 8 9 9 16 17 22 26 31	5 13 8 5 14 84 81 5 32 23 49 8 4 4 4 11 81 17 37 24 33 76 55 83	19 24 17 19 81 130 92 19 105 101 140 81 30 119 171 81 136 37 171 105 171 160 165 123	23 34 33 110 140	3 3 3 3 3 6 5 4 4 3 3 3 3 3 3 3 3 3 3 3 3 3 3 3

### 3.4 Theoretical Peptide Binding Spectrum of the Population‐Prevalent BoLA‐I Alleles in African Cattle and Comparison With European Cattle Allele Binding Profiles

#### 3.4.1 Comparison of the Potential Breadth of Peptide Presentation Between African and European Cattle BoLA‐I Alleles

**FIGURE 5:**
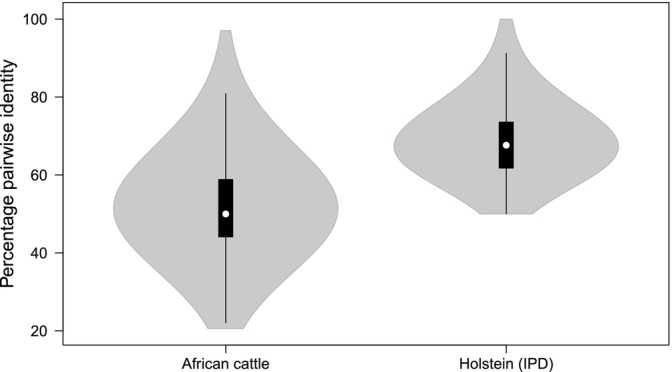
The distribution of the percentage pairwise identities among pocket forming residues of African cattle BoLA‐I alleles compared to Holstein allelic sequences present in the IPD. White circles show the medians; box limits indicate the 25th and 75th percentiles. Whiskers extend 1.5 times the interquartile range from the 25th and 75th percentiles. Polygons represent densi‐ty estimates of the data and extend to extreme values. The violin plot was generated using the R software vioplot package.

Anchor residues that determine whether a peptide will bind to a class I MHC molecule are found at position two (p2) and the C‐terminus (p9) of the canonical 9‐mer peptide, and these are accommodated by the class I MHC B and F pockets respectively. Additionally, p6 and p7 of a 9‐mer peptide sit in their respective C and E pockets. Other anchors are accommodated by the A and D pockets. We assessed the extent of diversity in the residue‐accommodating pockets [24] of BoLA‐I alleles in African and European cattle (Holstein‐Frisian) in order to illustrate differences in the potential breadth of peptide presentation. The analysis showed that the residue‐accommodating pockets are considerably more divergent in African cattle, with a mean percentage pairwise identity of 52.28 (± 11.39 SD). By comparison, the Holstein‐Frisian alleles in the immunopolymorphism database (IPD) exhibit a more limited repertoire of MHC specificity determining pocket residues, with a mean percentage pairwise identity of 82.53 (± 8.85 SD), potentially constraining the breadth of peptide presentation (Figure 5).

**FIGURE 6:**
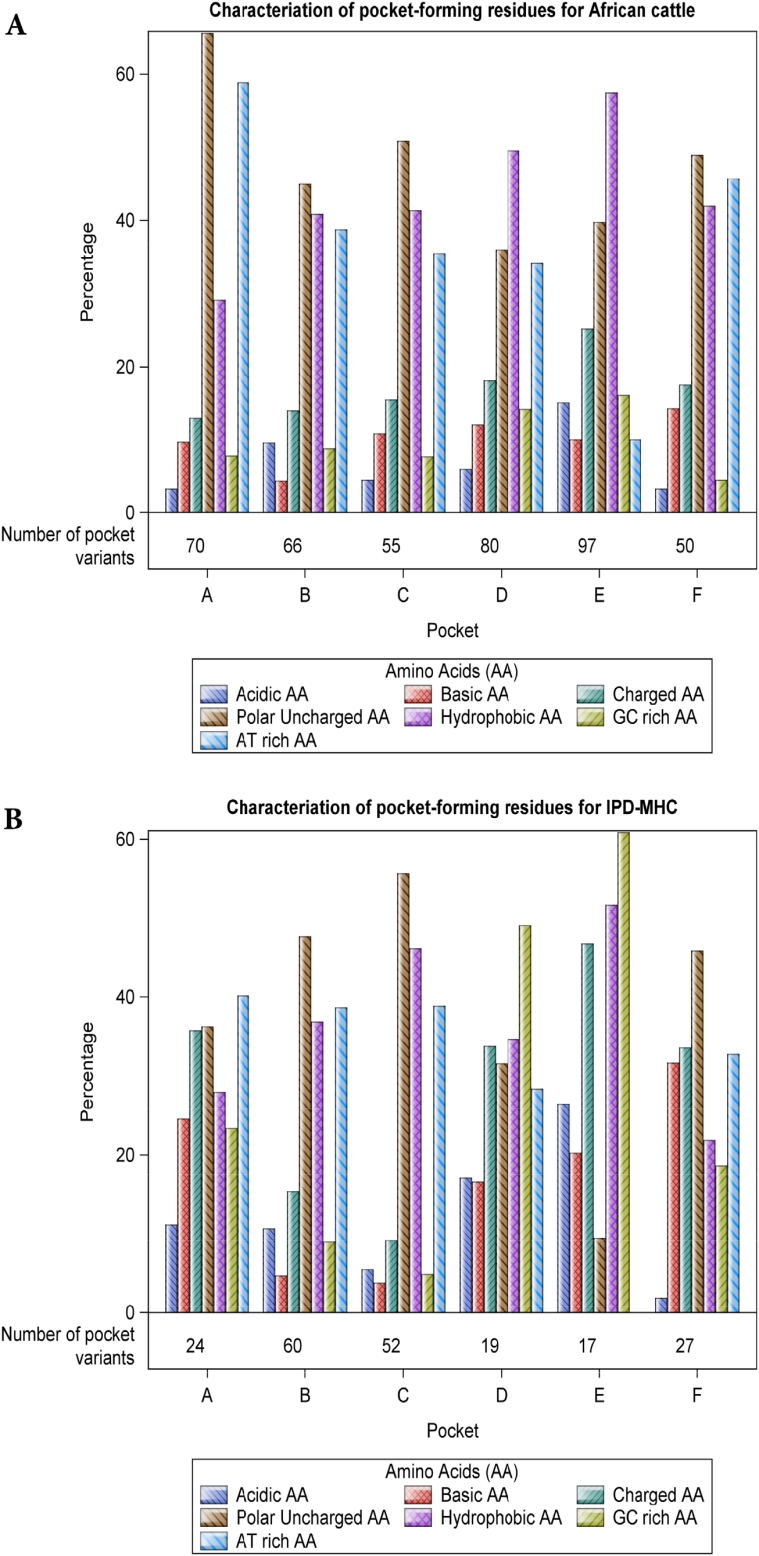
The physicochemical properties of the range of amino acids that line the primary and secondary anchor residue‐accommodating pockets of the population‐prevalent class I MHC alleles in African cattle (panel A) compared to European (IPD‐MHC) cattle alleles (panel B).

The polymorphisms located in the anchor pockets modulate both peptide selection for T‐cell recognition and the magnitude of the ensuing immune response. This structure–function relationship is dependent on physicochemical properties such as the hydropathy index and polarity of amino acids in positions key to the specificity of antigen binding. A comparison of the physicochemical properties of the variety of amino acids that line the primary and secondary anchor residue‐accommodating pockets of the African and European class I MHC alleles is shown in Figure 6.

#### 3.4.2 Divergence in the Pseudo‐Sequence Defining the BoLA‐I Binding Cleft Between African and European Cattle Alleles

Proteolytic processing of intracellular antigens results in a large pool of potential peptide ligands. However, only a limited number of peptides, termed the immunopeptidome, complement the polymorphic MHC specificity determining pockets in a way that leads to high‐affinity binding and efficient presentation. Three‐dimensional peptide–MHC structures have enabled identification of a 34 amino acid pseudo‐sequence, which describes positions of the class I MHC protein sequence critical to peptide binding as evidenced by the contact residues being within 4.0 A° of the peptide. This pseudo‐sequence encoding method is used to represent the binding context during the training of pan‐specific state‐of‐the‐art machine learning algorithms for MHC–peptide binding affinity predictions.

Only 18 of the 87 population‐prevalent African cattle BoLA‐I alleles were found to share a complete or very close to complete overlap (pseudo‐sequence distance < 0.05) with European cattle BoLA‐I alleles when considering the pseudo‐sequence defining the BoLA‐I binding cleft. The African taurine and indicine class I MHC sequences with close pseudo‐sequence distances to the known European *B. taurus* alleles, and therefore likely to have overlaps between their peptide‐binding repertoires, are shown in Table 3.

**TABLE 3:** African taurine and indicine BoLA‐I sequences with close pseudo‐sequence distances (< 0.05) to known European *Bos taurus* alleles and therefore likely to have overlapping peptide‐binding repertoires.

European *B. taurus* BoLA‐I	African cattle BoLA‐I	Pseudo‐sequence distance
BoLA‐1:019.01	6	0.000
BoLA‐2:012.01	8	0.027
BoLA‐2:048.01	24	0.000
BoLA‐2:048.01	29	0.038
BoLA‐2:008.01	51	0.000
BoLA‐1:023.01	65	0.000
BoLA‐T2b	67	0.026
BoLA‐3:002.01	73	0.000
BoLA‐gb1.7	81	0.017
BoLA‐4:024.01	85	0.000
BoLA‐2:012.01	87	0.000
BoLA‐2:016.01	97	0.000
BoLA‐gb1.7	105	0.000
BoLA‐gb1.7	123	0.000
BoLA‐gb1.7	130	0.000
BoLA‐gb1.7	133	0.000
BoLA‐gb1.7	136	0.017
BoLA‐gb1.7	140	0.000

#### 3.4.3 The African Cattle BoLA‐I Alleles Represent Five Novel Predicted Peptide Binding Specificities When Compared to European Cattle Alleles

**FIGURE 7:**
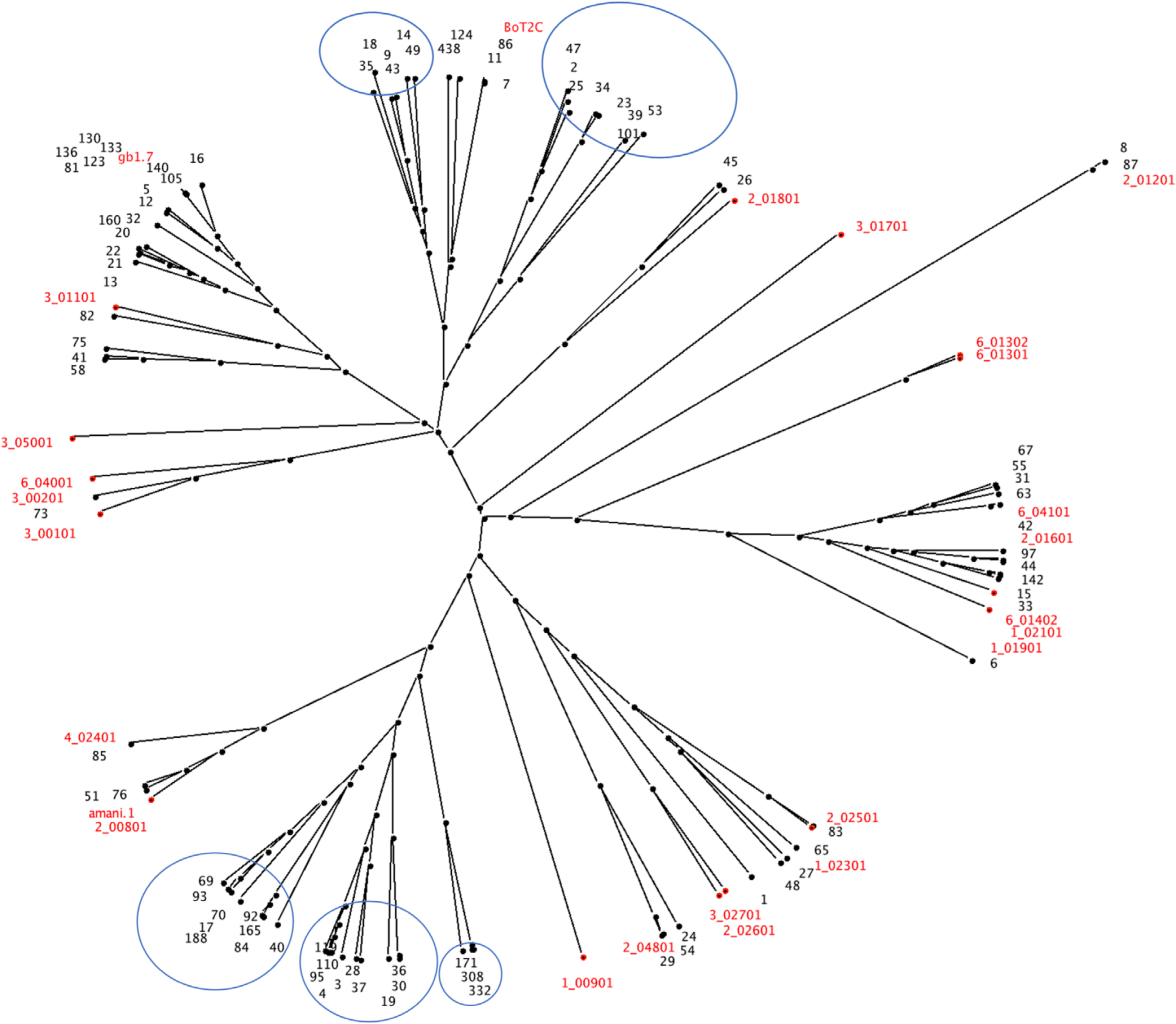
Class I MHC distance tree comparing the predicted peptide‐binding specificities of African (black branch labels) and European (red branch labels) cattle alleles. Branch support and consensus tree calculations were based on 1000 bootstrap replicates. Alleles with similar predicted peptide binding specificities branch together in groups or clusters (supertypes) and the closer two alleles branch, the larger the overlap between their predicted peptide‐binding repertoires. The novel specificity clusters are circled. The African cattle BoLA‐I alleles representing the five predicted novel peptide binding specificities are circled in blue. Representative logos illustrating the predicted peptide‐binding motifs for the five novel specificities are shown in Figure 8.

**FIGURE 8:**
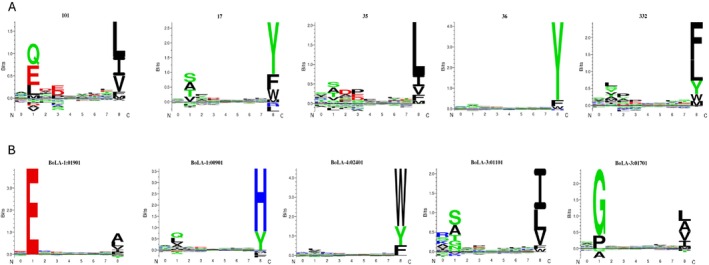
Representative logos illustrating the predicted peptide‐binding motifs for the five novel specificities (panel A) and the typical peptide binding motif for each of the other supertypes represented by European cattle BoLA‐I alleles (panel B). The height of each stack of symbols (*y*‐axis) represents information content (the level of amino acid conservation) in each position; the relative frequency of a particular amino acid at that position is represented by the individual height of the amino acid symbol and underrepresented amino acids.

We used NetMHCpan‐4.1, a machine learning‐based MHC ligand prediction tool, trained on eluted MHC‐bound peptides and in vitro binding assay datasets, to predict 9‐mer binding motifs for the population‐prevalent class I alleles in African cattle. The ligand prediction accuracy of the latest iteration of NetMHCpan, combined with methods that derive correlations between NetMHCpan predicted peptide binding affinities, is sufficiently discriminatory to answer questions on theoretical peptide binding spectra. We therefore performed functional clustering of African cattle class I MHC molecules based on their NetMHCpan‐4.1 predicted 9‐mer peptide binding motifs. A high correlation of the binding affinities of any two MHC molecules for the same set of peptides indicates similar binding preferences and thus a large functional overlap, whereas a low correlation indicates distinct binding preferences. Correlations between predicted binding affinities of the population‐prevalent class I MHC alleles were subsequently used to construct a functional distance tree. Immunopetidomic analysis has revealed the peptide‐binding specificities for the following European *B. taurus* class I MHC alleles: 1:009.01, 1:019.01, 1:021.01, 1:023.01, 2:008.01, 2:012.01, 2:016.01, 2:018.01, 2:025.01, 2:026.01, 2:048.01, 3:001.01, 3:002.01, 3:011.01, 3:017.01, 3:027.01, 3:050.01, 4:024.01, 6:013.01, 6:013.02, 6:014.02, 6:040.01, 6:041.01 and T2C [25]. These alleles were included in the analysis for the purposes of indicating likely differences in peptide presentation between the African and European cattle class I MHC alleles. As shown in Figure 7, putatively functionally similar class I MHC variants are sorted into ‘supertypes’ that revealed five novel specificities whose representative predicted peptide‐binding motifs are illustrated by the logos in Figure 8.

## 4 Discussion

Unlike in humans where three classical class I loci, HLA‐A, ‐B and ‐C, are present on all MHC haplotypes, six classical class I MHC genes are present in cattle and not all animals express alleles derived from the full complement of the six classical loci [26]. An additional complexity in cattle MHC genetics therefore arises from configuration variation, which describes a phenomenon in which the six classical class I MHC genes have variable haplotype structures, with usually one, two, or three of the genes present and expressed, in a variety of combinations on different haplotypes [14]. Given this complexity of cattle MHC genetics, it is not surprising that it has taken many years to generate a reference list of alleles and genes carried by the highly inbred Holstein breed. African cattle are not only outbred, but in some cases represent admixtures of *B. indicus* and *B. taurus* [27].

This is the first study, to the best of our knowledge, to identify fully phased class I MHC alleles spanning multiple functional domains (exons 1–7) in African cattle. The analysis is therefore important in supplementing the data from the very limited number of earlier NGS‐based studies that have catalogued polymorphisms in exons 2 and 3 of African cattle class I MHC transcripts [9, 15, 28]. These two previous studies were based on the illumina and 454 pyrosequencing technologies. For these short‐read sequencing approaches, after discarding sequences categorised as of insufficient quality by read filtering algorithms, the lengths of retained reads are typically insufficient to allow phasing of exon 2 and 3‐derived sequences, so these are typically analysed separately or by assembly of partial sequences generated from two separate amplicons (if sequence read length allows for this).

The accuracy of consensus base‐calls (99.9%), enabled by the improved PacBio CCS mode, provides a good basis for achieving high allelic resolution. This is because the length of reads commonly reached by PacBio far exceeds the length of the class I MHC transcript, avoiding potential assembly errors. However, despite the several full passes around the closed loop, PacBio libraries are not error‐free and will still contain some inaccurate reads. The hybrid filtering method that we used initially involved the use of DADA2, a stringent algorithm that has found application in the inference of microbial community structure and, more recently, in MHC genetics [29]. Subsequent implementation of the filtering algorithm described by Sommer et al. (2013), ensured further noise removal from the CCS reads [20]. One of the key features of DADA2 is that it requires an input file with the reliability of each base‐call in the reads specified (through base quality scores). This feature allows the parameterization of the dataset‐specific error model to benefit from the incorporation of quality scores, in contrast to methods that do not incorporate quality information in post‐filtering analysis. DADA2 additionally incorporates quantitative abundances in error models and not just abundance ranks. These attributes make it highly useful in MHC research. It is also important to bear in mind that although the class I MHC data available for European *B. taurus* is better than for African cattle, it is still incomplete. Consequently, high‐throughput technologies, such as that developed for this study, can find application across a broad spectrum of cattle populations to generate more comprehensive datasets.

Using this approach, we have defined fully phased, population‐prevalent class I MHC variants in East African cattle, generating data that can contribute to improved understanding of pathogen resistance and evidence‐based approaches to vaccine design. This study focused on Ankole and zebu cattle populations that are representative *B. taurus* and *B. indicus* breeds of some of the cattle populations in the area of Africa in which ECF is prevalent. As a major pathogen against which CD8+ T‐cell responses are critical for protection, assessing the diversity of class I MHC repertoires and the functional implications of this diversity is a prerequisite for rational vaccine development. A key finding was the observation that the most prevalent class I MHC variants are similarly transcribed in both African *B. indicus* (zebu) and *B. taurus* (Ankole) cattle (Figure 5). There are at least two, not necessarily mutually exclusive, possible explanations for the sharing of prevalent class I MHC alleles by African indicine and taurine cattle. Firstly, these subpopulations are under similar pathogen‐driven selective pressure, which may have shaped convergence of MHC‐I functional diversity. Secondly, although African *B. indicus* and *B. taurus* cattle are believed to represent two sub‐species of domestic cattle that diverged from a common ancestor between 200 000 and 1 million years ago, studies of mitochondrial DNA have revealed substantial mixing of the two subspecies, to the extent that all mitochondrial DNA is of taurine origin [30]. Furthermore, Y chromosome analysis has confirmed a progressive introgression of zebu ( *B. indicus* ) haplotypes into many African taurine populations [31]. The existing African cattle populations may therefore have inherited shared loci underpinning MHC diversity from both sub‐species through domestication and subsequent intermixing.

Because of the extensive divergence at the classical genes of the BoLA‐I complex in African cattle, we searched for evidence of elevated posterior probabilities of positive selection, based on dN/dS ratios under a Bayesian population genetics framework. Maximum‐likelihood analysis of evolutionary pressures acting on domains involved in the reciprocal coevolutionary arms race with pathogens revealed overwhelming evidence for the action of natural selection (ω > 1), potentially driven by repeated episodes of pathogen‐driven selective sweeps. African cattle are exposed to a very high diversity and intensity of disease challenge. In fact, certain pathogens may still be in the process of differentially shaping the MHC diversity of different cattle populations today. As a leading cause of cattle deaths in sub‐Saharan Africa, *T. parva* perhaps exerts particularly strong selection on the classical genes of the BoLA‐I complex, given its genetic complexity, coupled with its ability to undergo ‘sexual’ recombination, allowing for extensive genetic and antigenic diversity [6, 32]. In addition to the evidence demonstrating that MHC class I‐restricted CTLs are required to control *T. parva* infection, there is also evidence of extremes in the *T. parva* susceptibility spectrum. At one extreme of the spectrum are the non‐indigenous cattle (e.g., *B. taurus* , Holstein), introduced into Africa over the last 100 years which often succumb to *T. parva* infection, even in well‐resourced ranches despite the widespread use of acaricidal dipping. At the other extreme are Cape buffalo ( *Syncerus caffer* ) that have evolved in tandem with *T. parva* and exhibit no obvious clinical symptoms. Indigenous cattle, that have evolved under endemic disease challenge, are in an intermediate state. The potential for on‐going pathogen‐driven selective pressure related to host defence, particularly in genes of the core ‘immunome’ whose products directly interact with the pathogen, is therefore greatest in African cattle.

Our subsequent analyses were primarily aimed at a comparison of likely differences in peptide presentation between African and European cattle BoLA‐I alleles. This included assessing the extent of diversity of the residues involved in pocket formation, which contribute to the peptide binding motifs, as this can reveal the potential breadth of peptide presentation in a population. The anchor residue accommodating pockets are the adaptive interface of pathogen recognition that dictate the sequence, hydropathy, polarity and length of peptides selected for immune recognition. Consequently, only a limited number of pathogen‐derived peptides complement the MHC specificity‐determining pockets in a way that leads to high‐affinity peptide binding and efficient presentation to CD8 T cells. Our analysis of the pocket‐forming residues in the population‐prevalent BoLA‐I alleles of African cattle revealed high levels of polymorphism, which contrasts with Holstein alleles in the IPD that exhibit a more limited repertoire of MHC specificity‐determining pocket residues, which potentially constrains the breadth of peptide presentation. It would also seem, on the basis of this finding, that the existing cattle MHC databases require further input from African and potentially other geographical cattle populations.

The fact that only 18 of the 87 population‐prevalent African cattle BoLA‐I alleles were found to share a complete (or very close to complete) overlap to European *B. taurus* BoLA‐I alleles when considering the pseudo‐sequence defining the BoLA‐I binding cleft raised the possibility that the African cattle alleles represented additional novel peptide binding specificities. The ligand prediction accuracy of the latest iteration of NetMHCpan, combined with methods that derive correlations between predicted peptide binding affinities, enabled us to answer questions on likely differences in peptide presentation between the African and European BoLA‐I sequences. This interrogation of potential differences in peptide binding specificities revealed that the fully phased African cattle class I MHC alleles do represent at least five novel specificities. This suggests that the T‐cell responses restricted by these novel specificities may be directed to a different set of epitopes/antigens. This might indicate that in terms of future design of multivalent vaccines based on induction of CD8 T‐cell‐mediated responses against any pathogen, ensuring that the vaccine includes epitopes that bind strongly to these additional specificities would be necessary to ensure broad protection.

*T. parva* antigens recognised by the protective cellular immune responses have been identified in Holstein cattle, with the aim of investigating their potential for developing a subunit vaccine. However, analysis of immune African taurine (Obara et al. 2016) and indicine cattle (Akoolo et al. 2008) for their ability to recognise these antigens has revealed that the current suite of candidate antigens is not recognised by immune bovine CD8+ T cells from these African breeds. The current study therefore addresses a key obstacle to rational vaccine development against a major pathogen of cattle in sub‐Saharan Africa by paving the way for the identification of candidate vaccine antigens. In particular, immunoprecipitation and de novo sequencing of the ensemble of *T. parva* peptides presented by the population‐prevalent BoLA‐I specificities that we have identified will be a critical step in the ability to evaluate whether different *T. parva* antigens are presented in the major vaccine target African and European cattle populations. These studies will exploit advantages afforded by a biological feature of *T. parva* , that is, the ability to exponentially expand *T. parva‐*infected cells in vitro and thus supply the core material needed for the immunopeptidomic studies, which for many other pathogens is a significant constraint on the application of the technology. Building on the sequencing of fully phased class I MHC alleles in relevant African cattle populations, the focus of future work will be to conduct such immunopeptidomic studies as part of our continuing efforts to develop novel vaccines against ECF.

## Author Contributions

Isaiah Obara, Richard Bishop and Ard Nijhof conceived the study. Isaiah Obara and Anne Nanteza conducted the field work. Isaiah Obara, Khawla Elati and Naftaly Githaka conducted the laboratory work. Isaiah Obara, Andreotti Sandro, Morten Nielsen and Timothy Conneley performed the bioinformatics analysis and interpretation of the data. All authors have read and approved the final manuscript.

## Conflicts of Interest

The authors declare no conflicts of interest.

## Data Availability

The data that support the findings of this study have been deposited to NCBI (GenBank accessions: PQ155945–PQ156032).
